# The Relationship between Inflation and Inflation Uncertainty. Empirical Evidence for the Newest EU Countries

**DOI:** 10.1371/journal.pone.0091164

**Published:** 2014-03-14

**Authors:** Daniela Viorica, Danut Jemna, Carmen Pintilescu, Mircea Asandului

**Affiliations:** Faculty of Economics and Business Administration, Alexandru Ioan Cuza University of Iasi, Iasi, Romania; Cinvestav-Merida, Mexico

## Abstract

The objective of this paper is to verify the hypotheses presented in the literature on the causal relationship between inflation and its uncertainty, for the newest EU countries. To ensure the robustness of the results, in the study four models for inflation uncertainty are estimated in parallel: ARCH (1), GARCH (1,1), EGARCH (1,1,1) and PARCH (1,1,1). The Granger method is used to test the causality between two variables. The working hypothesis is that groups of countries with a similar political and economic background in 1990 and are likely to be characterized by the same causal relationship between inflation and inflation uncertainty. Empirical results partially confirm this hypothesis.

**Jel Classification:**

C22, E31, E37.

## Introduction

After 1990, in almost all of the newest EU countries was an evolution of inflation after a similar pattern, involving a high level of price volatility. In the first phase, these countries made an effort to decrease inflation, and this objective was achieved in the second part of the ‘90 s. Starting with 2003–2005, however, the inflation returned in various forms in some of the analysed countries, and the economic crisis established after 2008 has required extra effort to keep the increase of prices under control.

For the newest EU countries, the reduction of inflation became a major goal of economic policy, namely aiming to reach nominal and real convergence towards the EU integration. The economic and social costs generated by high inflation made the price stability, insured through a reduced and stable inflation, become the most important goal of the monetary policy of these countries.

The high inflation rates registered in the analysed countries in the first years of the ‘90 s also accentuated the uncertainty about future inflation, which can affect the financial markets by raising the long-term interest rates and lead to uncertainty about other economic variables (wages, tax rates). When inflation is high, there is political pressure for its reduction. Under these circumstances, future monetary policy will be unpredictable for the public, and therefore uncertainty about inflation will rise. In the case of the newest EU countries, the analysis of the relationship between inflation and inflation uncertainty holds special interest in the context of the need to insure sustainable economic growth. Regardless of the price of the stabilization strategy adopted in all the analysed countries, the relationship between inflation and inflation uncertainty remains an important research topic and brings useful results for the decision on economic policy and also for the business environment in order to have efficient and informed management decisions regarding future investments.

In the paper we have analyzed the relationship between inflation and inflation uncertainty for the newest EU countries that adhered in 2004, 2007 and 2013, namely Cyprus, the Czech Republic, Estonia, Hungary, Latvia, Lithuania, Malta, Poland, Slovakia, Slovenia, Bulgaria, Romania and Croatia. These countries have not only a process of common integration in the EU but also a past with important political and economic changes. More precisely, we would like to test the Friedman-Ball as well as the Cukierman-Meltzer hypotheses, and their reverse hypotheses, the Pourgerami-Maskus and respectively the Holland hypotheses. The Friedman-Ball hypothesis states that an increase in inflation will lead to more uncertainty about inflation. The Cukierman and Meltzer hypothesis states that when uncertainty about inflation increases, it causes high rates of inflation. By means of the empirical study performed, we will verify the causality relationships between inflation and inflation uncertainty for these countries.

In comparison with previous studies, this paper has analysed the newest EU countries considering their recent history, namely their political and economic background. We have grouped the analysed countries in five categories: the Baltic countries, which are the former Soviet Union countries (Estonia, Latvia, Lithuania); the group of countries which implemented early economic reforms (Hungary, Poland, Czech Republic, Slovakia); countries of the former Yugoslavia (Croatia, Slovenia); countries with late economic reforms (Romania, Bulgaria); small open economies (Cyprus, Malta).

The working hypothesis is that groups of countries with a similar political and economic background in 1990 are likely to be characterized by the same causal relationship between inflation and inflation uncertainty. Empirical testing of this hypothesis brings useful information by allowing for a possible correlation between the type relationship between inflation and its uncertainty and the socio-economic context of each country. The results are addressed, on the one hand, for the economic and financial policy makers whose objective is to obtain long-term price stability, and, on the other hand, to the business environment for decisions regarding future investment and economic growth for the analysed countries.

The contribution of this study can be highlighted at two levels. One is methodological: for the chosen sample of countries, a statistically robust result was provided when it came to testing the causality between inflation and its uncertainty by the simultaneous analysis of several models that estimate inflation uncertainty and by using a data sample large and updated. The working hypothesis of this research represents the second contribution. That is, the causal relationships between inflation and inflation uncertainty are tested under the assumption that for each group of countries, there will be the same type of validated relationship. In comparison with other studies that analyze the causality relationship between inflation and inflation uncertainty, this present study does not take into account a possible effect of EU accession (there are studies showing that actually this effect is not significant, such as [1]), nor the effect of their membership in the euro area.

This paper is structured as follows: In Section 2 we present a literature review on this subject; Section 3 deals with aspects related to data series and applied methodology, mainly ARCH-GARCH models used for the estimation of conditional residual variances, such as measures of uncertainty; Section 4 presents our empirical study. In the first part of the empirical study, we have presented a statistic overview regarding the evolution of inflation of the newest EU countries, and in the second part we attempted to highlight our empirical results for the hypotheses tested. The last section comprises our main conclusions.

## Literature Review

The extensive body of literature regarding the relationship between inflation and inflation uncertainty dates back more than 30 years when Okun [2] found, for 17 OECD countries, a positive relationship between the inflation rate and inflation variability. After that, the Nobel lecture address of Friedman [3] on the real effects of inflation generated extensive debates in the literature. Friedman stated that an increase in inflation will lead to more uncertainty about inflation, an assumption later developed and confirmed by Ball [4]. Pourgerami and Maskus [5] and then Ungar and Zilberfarb [6] also studied the relationship between inflation and inflation uncertainty but found evidence that high inflation reduces the uncertainty about inflation.

Examining the other causal relationship, that the inflation rate is determined by inflation uncertainty, Cukierman and Meltzer [7] found support that when there is uncertainty about increases in inflation, it causes high rates of inflation. The same causality, but with a negative relationship between variables, was found by Holland [8].

In order to investigate the relationship between inflation and its uncertainty, considering all the causal effects between the variables, we can study four possible causal relationships for the two variables considered. In Table 1 we have presented the most significant contributions made for each type of causality.

**Table 1:** The investigated hypotheses.

Hypothesis	Sign of the causal relationship
**H1: Inflation Granger-causes inflation uncertainty**	
[3], [4]	+
[5], [6]	−
**H2: Inflation uncertainty Granger-causes inflation**	
[7]	+
[8]	−

The first hypothesis is the most investigated and has the strongest theoretical and empirical background, given the debates around the Nobel-awarded contribution of Friedman.

Friedman’s and Ball’s research findings emphasize a positive relationship between inflation and inflation uncertainty. They argue that when the inflation rate increases, the monetary authorities do not have a clear response, and this generates uncertainty about the future rate of inflation for the public, since the money supply growth cannot be predicted. On the contrary, Pourgerami and Maskus, as well as Ungar and Zilberfarb state that high inflation could lead to lower uncertainty regarding inflation, since in the case of increased inflation, more resources would need to be invested in order to accurately predict the future inflation rate, and this action would lower the uncertainty level.

Cukierman and Meltzer found support for a positive relationship between the two variables. They argue that increasing inflation uncertainty generates opportunistic behaviour from the policy authority, meaning they generate surprise inflation for the economic agents, in order to obtain output gains. On the opposite side, Holland found evidence of a negative relationship between the variables, suggesting that in the case of increased inflation uncertainty, the policymaker has stabilizing behaviour. This means that the monetary authority will reduce the money supply growth in order to reduce the negative welfare effects. Grier and Perry [9] suggest that the opportunistic or stabilizing behaviour of the monetary authorities is related to the level of central bank independence. The higher the level of central bank independence, the lesser the rate of inflation.

Evans [10], Grier and Perry [9] found empirical evidence to support the Friedman-Ball hypothesis. Evans analyzed the relationship between long-term inflation uncertainty and the inflation rate for the USA, and Grier and Perry investigated the relationship for G7 countries, using GARCH models to estimate the inflation uncertainty. Both studies offered strong evidence in support for the Friedman-Ball hypothesis and weak evidence in support for the Cukierman-Meltzer hypothesis.

In the UK, Fountas [11] and Kontonikas [12] investigated the relationship between the two variables and confirmed the Friedman-Ball hypothesis. Kontonikas analyzed the effect of inflation targeting policies on reducing inflation variability and found a negative impact of inflation targeting on long-run uncertainty.

For the E.U countries, there are several significant studies. Fountas, Ioannidis and Karanasos [13] employed E-GARCH models to estimate inflation uncertainty and found strong evidence to support the Friedman-Ball hypothesis and mixed evidence for the second investigated hypothesis for a sample of six EU countries. They suggest that the European Central Bank can lower inflation uncertainty by targeting inflation. Another important study is [14], who analyzed the relationship between inflation and inflation uncertainty for the Euro zone, using an AR-GARCH model for inflation. The results showed that, after the introduction of the Euro and with strong anti-inflation measures, empirical support was found for the Friedman-Ball hypothesis, suggesting that by focusing on long-run price stability a lower inflation uncertainty can be achieved.

Conrad and Karanasos [15] analyzed the dual long-memory behaviour of inflation in relation to inflation uncertainty using an ARFIMA-FIGARCH model. Their results showed that inflation raises inflation uncertainty for all countries analyzed, the USA, Japan and the UK. Thornton [16], who used a GARCH model to estimate the uncertainty, for a sample of 12 emerging economies as well as [17], who employed the GARCH models to estimate uncertainty for Germany, the Netherlands and Sweden, found the same results.

The two hypotheses were tested for the most recently adhered countries to the European Union. There are several studies that found empirical evidence for the investigated hypothesis. From those studies, three are more extensive – [18], [19] and [1] – who investigated the causal relationships between the two variables for a sample of countries, as presented in Table 2.

**Table 2:** Significant body of empirical evidence existent in the literature for the investigated hypotheses and countries.

Country	Research paper	Hypotheses/Signs/Lags	
		H 1	H 2
Poland	[18]	FB – 4, 8, 12	H – 4, 8, 12
	[19]	FB – 4,8, 12	H – 12
	[18]	FB – 4, 8, 12	H – 4
Romania	[30]	FB	–
	[19]	FB – 4, 8, 12	–
	[18]	FB – 12	H – 4
Bulgaria	[19]	FB	
	[30]	FB	–
	[16]	–	CM
Hungary	[1]	–	CM
	[18]	FB – 4, 8, 12	CM – 4, 8, 12
Latvia	[29]	FB – 5, 10, 20, 30	CM – 5, 10, 20, 30
	[18]	–	–
Lithuania	[1]	–	CM
	[19]	FB – 4, 8, 12	H – 4, 8, 12
Cyprus	[1]	–	–
Czech Rep.	[18]	FB	
	[19]	PM	
Malta	[1]	–	–
Slovakia	[18]	FB	H
Slovenia	[1]	–	CM
Estonia	[19]	PM – 4, 8, 12	–
Croatia	[18]	FB	H
	[1]	–	CM

*Note:* FB – Friedman-Ball, PM – Pourgerami-Maskus, H – Holland, CM – Cukierman-Meltzer.

For the first hypothesis, overwhelming evidence was found to support the Friedman-Ball hypothesis. For the second hypothesis, mixed results were obtained in the literature, given the fact that the analyzed countries have different financial policies and different lags in implementing structural reforms. Hence there are no evident patterns in the literature to support a certain type of causality for the sample of countries for the second hypothesis.

## Data and Methodology

In this study, for inflation, we have considered the monthly data for the Consumer Price Index (CPI), data provided by the International Financial Statistics (IFS). The analysis period is January 1990– December 2012. In compliance with the literature ([1], [18]) the data are transformed, and we obtained the variable: 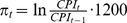. By these transformations we obtained the month-on-month percentage growth rates, which are usually annualized i.e. multiplied by a factor of 1200 to give the amount the series would grow in a year. Thus, we provide the most up-to-date growth rates, which are less biased than year-to-year growth rates.

According to the literature, inflation uncertainty is measured by means of the conditional variance which is obtained by means of an econometric model from the class of ARCH-GARCH heteroscedastic models. Various authors used models, such as ARCH(1), GARCH(1,1) ([20], [21]) or more complex models, such as EGARCH [19] and PARCH [22].

The approach of the empirical study involves the development of the following stages: testing the stationarity for the time series for each country; estimating an autoregressive model for inflation; choosing a heteroscedastic model that could estimate the conditional variances of inflation; testing the existence of a causality relationship between inflation and inflation uncertainty; identifying the sign of the correlation between these two variables, in case it exists.

Taking into account the limits of the most used stationarity test, the ADF (Augmented Dickey-Fuller) test, in this paper for testing the stationarity, we used in parallel other two tests: Phillips-Perron and KPSS (Kwiatkowski-Phillips-Schmidt-Shin) tests. If the tests do not allow the rejection of the hypothesis of the existence of a unit root (the non-stationarity property) the data series must be transformed by means of the operator difference until a stationary series is obtained.

For the stationary series an AR(p) autoregressive model was built. The order of this model takes values within 1 and 12, according to the monthly frequency of the available data. For the estimated models the significance of parameters is tested and out of all the possible models the model that admits the minimum value for the Akaike and Schwartz information criteria is chosen. An AR(p) model for inflation has the form:
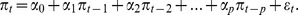



After the choice of the explanatory model of inflation for each country, a heteroscedastic model is estimated, enabling the estimation of inflation uncertainty. In this study, taking into account the limits and advantages suggested by the literature, we considered in parallel four models of the ARCH-GARCH class: ARCH(1), GARCH (1,1), EGARCH (1,1,1) and PARCH(1,1,1). All four models were estimated and tested and after verifying the traditional hypotheses regarding the errors, the best model was chosen with the help of the Akaike and Schwartz information criteria.

If we accept the known notation for the conditional variance, *h_t_* (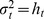), of an ARCH model of order *q*, this variable can be obtained as follows:
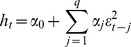
where 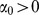 and 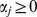, condition for *h_t_* to be always positive.

The ARCH (1) model is the simplest and has only two parameters for the equation of the conditional variance: 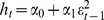.

In 1986, Bollerslev introduced the GARCH (p,q) generalized model for which the conditional variance has an equation which also takes into consideration the previous conditional variances:
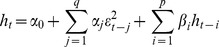



For the estimation of inflation uncertainty, in the studies from the literature a GARCH (1,1) model is usually used, which estimates the present conditional variances by means of the square errors and the variances from the previous moment: 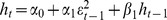.

The EGARCH model or the Exponential GARCH model establishes an exponential relationship between the present variances and the previous errors. Through the model introduced by Nelson [23], the inflation uncertainty has the form:
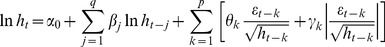



As it can be noticed from the above equation, the conditional variances are influenced both by the error values and by their sign. In this paper, we used an EGARCH (1,1,1) model, where the conditional variances are estimated by means of a model of the form:
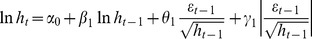



Another development of the GARCH models was performed by Taylor [24] and Schwert [25], who introduced the standard deviation of the GARCH model, where the standard deviation is modeled rather than the variance. This model, along with several other models, was generalized by Ding *et al*. [26] with the Power ARCH specification. In the Power ARCH model, the power parameter *δ* of the standard deviation can be estimated rather than imposed, and the optional *γ* parameters are added to capture asymmetry of up to order *r*:
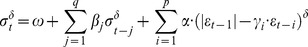



The Bollerslev [27] model sets *δ  = 2, γ  = 0*, and the Taylor [24] model sets *δ  = *1 and *γ  = 0*. Empirical estimates indicate that the power term is sample dependent and values of near 1 are common in the case of stock data [26], while for foreign exchange data the power term varies between 1 and 2 [28]. In this paper, we opted for a PARCH (1,1,1) type of model, which is estimated alongside the other three previous models.

The study of the causality between inflation and inflation uncertainty is performed by means of the Granger causality test that verifies to which extent a variable is explained through the addition of previous values for the other variable. Such a relationship is important in the case of inflation and inflation uncertainty, while the test allows for the verification of one of the two hypotheses formulated in the literature: inflation determines uncertainty or the other way round.

In order to test the first hypothesis, the relationship used is:
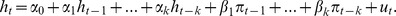



For this model we tested the hypothesis 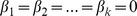, meaning that inflation does not Granger cause inflation uncertainty.

In the last part of the empirical study, after testing the two hypotheses by means of the Granger test, we established the sign of this relationship. To this aim, a VAR model was built for 4, 8 and 12 lags. The sign of the correlation is given by the sum of the coefficients for the variable inflation in the first hypothesis, while for the inflation uncertainty, for the second one, it is given by the equations in the VAR models. In this case, the VAR model is of the form:
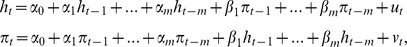
where *m* alternatively takes the values 4, 8 and 12.

## Empirical Study

This empirical study aims to test the relationship between inflation and inflation uncertainty for the 13 newest EU countries and the identification of the sign of this relation, in case there is one. In the first part of the study, we present the evolution of inflation rate for the countries of our sample. In the second part, we present our main results of the econometric estimations.

### 4.1 Inflation Dynamics for the Newest EU Countries

**Figure 1:**
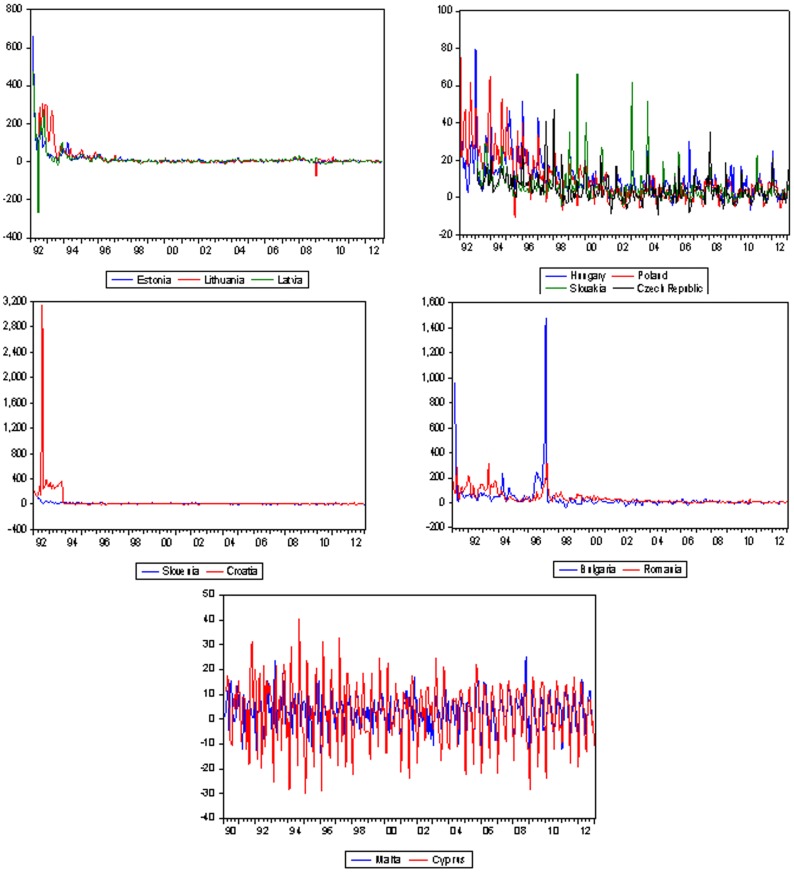
The inflation dynamics registered in the newest EU countries in 1990–2013.

The inflation dynamic for the newest EU countries, after 1990, using the annualized monthly growth rates of the Consumer Price Index, is presented in Figure 1. The evolution is displayed according to the five groups of countries mentioned in this paper.

The reforms adopted in the ‘90 s by the Central and Eastern European countries regarding price liberalization generated very high inflation. Following the measures of economic policy adopted after 1990, the yearly inflation rate in some countries exceeded 200%. For instance, in 1993 in Lithuania the inflation rate was 410% and in Romania the rate of inflation was 256.1%. The reformation and restructuring process of the economic system determined in many countries drastic real output falls during the early stages of transition. Today, these high rates of inflation have increased uncertainty about the ability of the authorities in these countries to ensure price stability.

The inflation analysis of the newest EU countries mentioned in this section highlighted similar dynamics for certain groups of countries, depending on their specific political and economic backgrounds that existed in the ‘90 s. In order to study whether between inflation and inflation uncertainty in these groups of countries there is the same type of causality relationship, we will estimate, in the next paragraph, the econometric model of the relation between these two variables.

### 4.2 Modeling the Relationship between Inflation and Inflation Uncertainty

In order to model the relationship between inflation and inflation uncertainty, the stationarity of data series was tested using the ADF, PP and KPSS tests. After testing the stationarity, we presented the equations estimated for the CPI as well as for inflation uncertainty. The testing of the existence of a relationship between inflation and inflation uncertainty was performed using the Granger-causality test. Finally, a VAR model was used to identify the sign of the relationship between the two variables.

#### 4.2.1. Testing the series stationarity

In a first stage, we tested the stationarity of the time series for each country. The stationarity tests applied are the Augmented Dickey-Fuller (ADF) and Phillips-Perron (PP) tests, for which the null hypothesis is the non-stationarity hypothesis as well as the Kwiatkowski-Phillips-Schmidt-Shin (KPSS) test, for which the null hypothesis is the stationarity hypothesis. After applying these tests, the results shown in Table 3 were obtained.

**Table 3:** Unit root tests.

Country	ADF	PP	KPSS
Bulgaria	−11.99	−12.03	0.90
Croatia	−6.19	−15.57	0.65
Cyprus	−4.43	−19.39	0.52
Czech Republic	−2.37	−12.37	1.43
Estonia	−7.37	−18.23	0.85
Hungary	−2.52	−11.97	1.95
Latvia	−3.88	−9.02	0.63
Lithuania	−9.77	−4.24	0.76
Malta	−7.00	−21.53	0.26
Poland	−7.43	−10.55	1.51
Romania	−5.18	−7.56	1.65
Slovakia	−2.90	−12.11	1.38
Slovenia	−5.56	−10.43	1.10

*Note*: A constant and 12 lagged difference terms are used for the Augmented Dickey-Fuller test. The MacKinnon critical value for the rejection of the unit root null hypothesis at the 1% significance level is −3.45. The KPSS critical values for the rejection of the unit root null hypothesis at the 1%, 5% and 10% significance levels are 0.739, 0.463 and 0.347, respectively.

When analyzing the results in Table 3, we may consider that we have strong reasons, for a significance level of 10%, to reject the non-stationarity hypothesis (existence of unit root), the time series being used in the modeling process without being changed.

#### 4.2.2. Modeling inflation

For the countries under study, the AR(p) models were estimated, where *p* is the order of the auto-regression models and takes values between 1 and 12. The results of the data processing are presented in Table 4.

**Table 4:** Econometric equations for inflation.

Country	Lags	AIC	SIC	Equation
Bulgaria	1,6	11.92	11.96	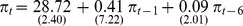
Croatia	1,2,3,6	13.33	13.44	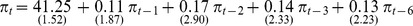
Cyprus	1,2,3,4,5,6,7, 8,9,10,11,12	6.95	7.03	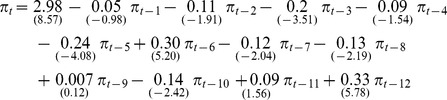
Czech Republic	1,6,12	6.37	6.43	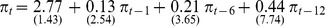
Estonia	1,2,3,6	7.52	7.59	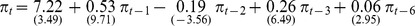
Hungary	1,4,8,12	6.99	7.06	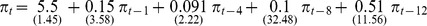
Latvia	1, 2, 12	6.68	6.74	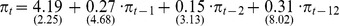
Lithuania	1, 3, 6, 12	7.59	7,67	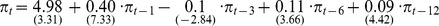
Malta	3,6,12	6.56	6.61	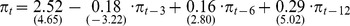
Poland	1, 12	7.18	7.22	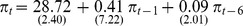
Romania	1, 3, 12	9.70	9.75	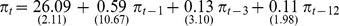
Slovakia	1,12	6.93	6.97	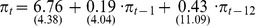
Slovenia	1, 6, 12	6.59	6.64	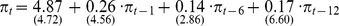

Following the estimating and testing of several auto-regression models, the best models were identified, being presented in the table above, using the Akaike and Schwartz information criteria. These models represent the basis for building the ARCH-GARCH heteroscedastic models from the next stage, which will measure inflation uncertainty.

#### 4.2.3. Modeling inflation uncertainty

The models estimated for measuring inflation uncertainty are presented in Table 5.

**Table 5:** Econometric equations for inflation uncertainty.

Country	Model	AIC	SIC	Equation	ARCH LM(1)	ARCH LM(4)	DW
Bulgaria	EGARCH	8.91	9.01	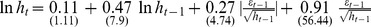	1.09 (0.29)	0.77 (0.54)	2.01
Croatia	GARCH	12.31	12.42	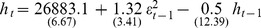	0.32 (0.57)	0.35 (0.84)	1.48
Cyprus	GARCH	6.81	6.99	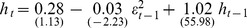	0.02 (0.88)	0.28 (0.88)	2.1
Czech Republic	EGARCH	6.15	6.27	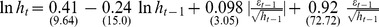	0.62 (0.43)	0.97 (0.42)	1.85
Estonia	GARCH	6.7	6.81	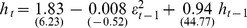	1.0 (0.30)	0.41 (0.79)	1.93
Hungary	GARCH	6.55	6.66	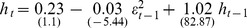	0.28 (0.59)	0.41 (0.79)	2.17
Latvia	GARCH	6.61	6.72	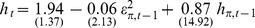	1.63 (0.20)	2.10 (0.08)	1.78
Lithuania	EGARCH	7.51	7.37	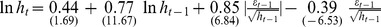	0.29 (0.58)	0.21 (0.93)	2.48
Malta	GARCH	6.56	6.65	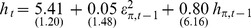	0.59 (0.44)	0.45 (0.63)	2.25
Poland	GARCH	6.29	6.37	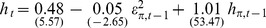	2.19 (0.13)	1.09 (0.35)	1.90
Romania	EGARCH	8.16	8.61	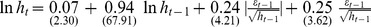	0.00 (0.96)	0.48 (0.74)	1.94
Slovakia	EGARCH	6.49	6.60	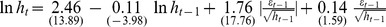	0.25 (0.61)	0.36 (0.83)	1.64
Slovenia	GARCH	6.51	6.61	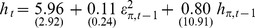	0.11 (0.73)	0.09 (0.98)	1.43

*Note*: The ARCH LM tests confirm the alienation of the heteroscedastic component in the models built for inflation uncertainty while the Durbin Watson test confirms the lack of series correlation. For the normality hypothesis it was considered that the feature is asymptotically reached for a sufficiently high amount of data in the sample.

For the estimation of inflation uncertainty four heteroscedastic models were used for each country in the sample: ARCH (1), GARCH(1,1), EGARCH(1,1,1) and PARCH (1,1,1). By means of these models, the conditional variances were estimated measuring the inflation uncertainty for each country. The best model was selected using the Akaike and Schwartz information criteria. The equations estimated for inflation uncertainty are presented in Table 5.

For the five countries under analysis the EGARCH(1,1,1) model was validated: Bulgaria, Czech Republic, Lithuania, Romania and Slovakia. For the rest of the countries, the GARCH (1,1) model was validated. The PARCH(1,1,1) model was not validated for any of the countries analyzed.

#### 4.2.4 The Granger causality test

In the subsequent stage we applied the Granger causality test in order to verify the relationship between inflation and inflation uncertainty. The results of the data processing are presented in Table 6.

**Table 6:** VAR Granger Causality Tests.

Country	Null Hypothesis	4 lags	8 lags	12 lags
Bulgaria	Inflation does not Granger Cause Inflation Uncertainty	1852.7	1934.6	1968.8
		(0.00)	(0.00)	(0.00)
		(+)	(−)	(−)
	Inflation Uncertainty does not Granger Cause Inflation	84.9	115.7	155.2
		(0.00)	(0.00)	(0.00)
		(−)	(−)	(−)
Croatia	Inflation does not Granger Cause Inflation Uncertainty	5899.6	7565.7	9340.2
		(0.00)	(0.00)	(0.00)
		(+)	(+)	(+)
	Inflation Uncertainty does not Granger Cause Inflation	30.9	52.8	76.1
		(0.00)	(0.00)	(0.00)
		(−)	(−)	(−)
Cyprus	Inflation does not Granger Cause Inflation Uncertainty	7.6	6.9	13.8
		(0.10)	(0.53)	(0.31)
		(+)	(+)	(+)
	Inflation Uncertainty does not Granger Cause Inflation	6.9	13.9	13.9
		(0.10)	(0.08)	(0.30)
		(+)	(+)	(+)
Czech Republic	Inflation does not Granger Cause Inflation Uncertainty	23.0	30.0	34.2
		(0.00)	(0.00)	(0.00)
		(+)	(+)	(+)
	Inflation Uncertainty does not Granger Cause Inflation	26.5	27.8	29.0
		(0.00)	(0.00)	(0.00)
		(+)	(+)	(+)
Estonia	Inflation does not Granger Cause Inflation Uncertainty	205.6	268.2	226.0
		(0.00)	(0.00)	(0.00)
		(−)	(−)	(−)
	Inflation Uncertainty does not Granger Cause Inflation	12.3	52.8	66.8
		(0.01)	(0.00)	(0.00)
		(+)	(+)	(+)
Hungary	Inflation does not Granger Cause Inflation Uncertainty	22.2	120.2	187.4
		(0.00)	(0.00)	(0.00)
		(−)	(−)	(−)
	Inflation Uncertainty does not Granger Cause Inflation	12.2	55.1	51.6
		(0.01)	(0.00)	(0.00)
		(+)	(+)	(+)
Latvia	Inflation does not Granger Cause Inflation Uncertainty	6.00	11.52	31.94
		(0.19)	(0.17)	(0.00)
		(−)	(−)	(−)
	Inflation Uncertainty does not Granger Cause Inflation	11.59	15.41	45.72
		(0.02)	(0.05)	(0.00)
		(−)	(+)	(−)
Lithuania	Inflation does not Granger Cause Inflation Uncertainty	73.33	94.11	121.13
		(0.00)	(0.00)	(0.00)
		(−)	(−)	(−)
	Inflation Uncertainty does not Granger Cause Inflation	7.31	8.78	28.30
		(0.12)	(0.36)	(0.00)
		(+)	(+)	(+)
Malta	Inflation does not Granger Cause Inflation Uncertainty	8.94	18.92	18.58
		(0.06)	(0.01)	(0.09)
		(−)	(+)	(+)
	Inflation Uncertainty does not Granger Cause Inflation	3.55	6.40	17.93
		(0.46)	(0.60)	(0.11)
		(−)	(−)	(−)
Poland	Inflation does not Granger Cause Inflation Uncertainty	45.86	64.46	56.45
		(0.00)	(0.00)	(0.00)
		(−)	(−)	(+)
	Inflation Uncertainty does not Granger Cause Inflation	24.05	35.97	24.54
		(0.00)	(0.00)	(0.01)
		(+)	(+)	(+)
Romania	Inflation does not Granger Cause Inflation Uncertainty	576	637	796
		(0.00)	(0.00)	(0.00)
		(+)	(+)	(+)
	Inflation Uncertainty does not Granger Cause Inflation	6.51	14.68	41.41
		(0.16)	(0.06)	(0.00)
		(+)	(−)	(−)
Slovakia	Inflation does not Granger Cause Inflation Uncertainty	58.84	91.88	43.88
		(0.00)	(0.00)	(0.00)
		(+)	(+)	(+)
	Inflation Uncertainty does not Granger Cause Inflation	0.44	21.06	96.50
		(0.97)	(0.00)	(0.00)
		(+)	(+)	(−)
Slovenia	Inflation does not Granger Cause Inflation Uncertainty	1.21	6.61	8.07
		(0.87)	(0.57)	(0.77)
		(−)	(+)	(−)
	Inflation Uncertainty does not Granger Cause Inflation	26.74	23.89	18.34
		(0.00)	(0.00)	(0.10)
		(−)	(−)	(−)

The results from table 6 represent the value of the statistics F and of the probability associated to it, used in the Granger test. These results highlight the existence or non-existence of a significant correlation between inflation and inflation uncertainty. The sign of the relationship between inflation and inflation uncertainty, a positive or negative relation, for 4, 8 and 12 lags is presented in table 6. The sign was identified by means of a VAR model between inflation and conditional variances.

The study conducted highlighted a positive relationship between inflation and inflation uncertainty, for Croatia, Cyprus, the Czech Republic, Romania and Slovakia, and a negative relationship for Estonia, Hungary, Latvia and Lithuania. The Friedman-Ball or Pourgerami and Maskus hypotheses were confirmed only for these countries.

As for the Cukierman-Meltzer or Holland hypotheses, the empirical results obtained highlighted a positive relationship between inflation uncertainty and inflation for Cyprus, the Czech Republic, Estonia, Hungary, Lithuania and Poland. Empirical evidence was found for a negative relationship between these two variables for Bulgaria, Croatia, Malta and Slovenia. These results are presented in Table 7.

**Table 7:** The results obtained by the five groups of countries for the investigated hypotheses.

Groups of countries	Countries	H1	H2
	Latvia	PM	–
Former Soviet Union	Lithuania	PM	CM
	Estonia	PM	CM
	Hungary	PM	CM
Early Economic reforms	Poland	–	CM
	Czech Rep	FB	CM
	Slovakia	FB	–
Former Yugoslavia	Slovenia	–	H
	Croatia	FB	H
Late economic reforms	Romania	FB	–
	Bulgaria	–	H
Small open economies	Cyprus	FB	CM
	Malta	–	H

These results highlight the fact that for certain groups of countries with a similar economic and political backgrounds in the ‘90 s there is empirical evidence of dominant behaviour regarding the causality relationships between inflation and inflation uncertainty.

For the three Baltic countries, Latvia, Lithuania and Estonia, high inflation has had a positive effect on inflation uncertainty. For Estonia, the sign of the relationship between inflation and inflation uncertainty can be explained by the high credibility of its currency board, while for the two other countries, the resources invested in order to break from the influence of Russia and from the Russian economy, together with efforts made to adjust and direct towards the European market have determined a decrease of the uncertainty about future inflation.

The second group of countries, consisting of Hungary, Poland, the Czech Republic and Slovakia is characterized by the fact that the uncertainty about inflation has a direct influence on inflation. In periods of high inflation, in the pre-EU accession period, high uncertainty generated high levels of inflation, but after the accession, these high-performance economies, especially the Polish economy, have experienced the reverse: low uncertainty that caused low levels of inflation.

The empirical evidence found for Slovenia and Croatia highlights that high uncertainty causes low inflation. For these countries, the policy authorities had a stabilizing behaviour, reducing the money supply growth in order to reduce the negative welfare effects. Also, their Central Banks have low independence levels, which, according to [9], correspond to stabilizing behaviour from the policymakers.

For the other two groups of countries, one formed by Romania and Bulgaria, the other by Malta and Cyprus, there are no empirical evidence to support our working hypothesis that for countries with similar economic and political backgrounds we have a dominant type of relationship between inflation and inflation uncertainty. Even if Bulgaria and Romania were characterised by a similar economic and political situation at the beginning of the transition period, they have experienced different lags and difficulties in implementing structural reforms. For instance, Bulgaria suffered a deep financial and monetary crisis, followed by a period of hyperinflation and a sharp devaluation of its national currency in 1997. In Romania, due to the price liberalization that started in 1991, there was a significant increase in the price of consumption and the highest level of inflation was registered in 1993, when the consumption prices had a yearly average variation of 256.1%.

Cyprus and Malta are very small open economies and have very specific features, with political backgrounds very different from the other EU countries in the sample and very different from each other. The political instability in Cyprus, along with its dependence on oil as its primary source of energy and the inconsistency of oil prices on international markets has generated an increased state of uncertainty for Cyprus. Malta, on the other hand, has achieved political stability by adopting a policy of neutrality but keeping close relations with the EU and USA. Also, Malta’s financial services’ industry is fairly stable and has managed to avoid the European financial crisis, its debt being mostly domestic. Thanks to this situation of stability, the uncertainty has had a positive effect on inflation.

## Conclusions

The reform measures of the economies of Central and Eastern European countries adopted in the ‘90 s generated high inflation rates. The insurance of price stability has thus become the main goal of the monetary policy in these countries, especially aimed at reaching nominal and real convergence towards EU integration.

Thirteen European countries had adhered to the European Union in 2004, 2007 and 2013. Considering the economic and political background of these countries in the ‘90 s, which was the start of the transition period to a market economy for most of these countries, we considered five different groups of countries with similar characteristics. The working hypothesis for this study is that groups of countries with similar political and economic backgrounds in ‘90 s are likely to be characterized by the same causal relationship between inflation and inflation uncertainty. We found empirical evidence to partially support this hypothesis. The results highlighted a dominant behavior regarding the causality relationship between inflation and its uncertainty for groups of countries with similar economic and political backgrounds in the ‘90 s. These groups are: the Baltic countries, the group of countries which implemented early economic reforms and the former countries of Yugoslavia.

The dominant behaviour for the Baltic countries is that high inflation has had a positive effect on inflation uncertainty. The group of countries with early economic reforms are characterised by the fact that the uncertainty about inflation has had a direct influence on inflation. In the case of Croatia and Slovenia, high uncertainty causes low inflation. For the other two groups of countries, no empirical evidence was found to support a certain type of behaviour regarding the relationship between inflation and its uncertainty, since the countries in these groups have had different economic and political patterns after 1990.

The results of our empirical study could be useful for the decision-makers in implementing or adjusting their monetary policy. For example, if the Friedman-Ball and/or Cukierman-Meltzer hypotheses are confirmed, then the monetary authorities could consider an inflation targeting policy in order to reduce inflation uncertainty and its negative effect on inflation, and consequently on economic growth.

A future direction of our research regards the causality analysis of the relationship between inflation and inflation uncertainty for these groups of countries according to the monetary strategy adopted by the monetary authorities to assure price stability: inflation targeting or exchange rate policy.
